# Replication and Shedding of MERS-CoV in Upper Respiratory Tract of Inoculated Dromedary Camels

**DOI:** 10.3201/eid2012.141280

**Published:** 2014-12

**Authors:** Danielle R. Adney, Neeltje van Doremalen, Vienna R. Brown, Trenton Bushmaker, Dana Scott, Emmie de Wit, Richard A. Bowen, Vincent J. Munster

**Affiliations:** Colorado State University, Fort Collins, Colorado, USA; (D.R. Adney, V.R. Brown, R.A. Bowen);; National Institutes of Health, Hamilton, Montana, USA (N. van Doremalen, T. Bushmaker, D. Scott, E. de Wit, V.J. Munster)

**Keywords:** MERS-CoV, coronavirus, Middle East Respiratory Syndrome, dromedary, camels, inoculation, infection, upper respiratory tract, transmission, experimental, zoonoses, viruses, zoonosis

## Abstract

In 2012, a novel coronavirus associated with severe respiratory disease in humans emerged in the Middle East. Epidemiologic investigations identified dromedary camels as the likely source of zoonotic transmission of Middle East respiratory syndrome coronavirus (MERS-CoV). Here we provide experimental support for camels as a reservoir for MERS-CoV. We inoculated 3 adult camels with a human isolate of MERS-CoV and a transient, primarily upper respiratory tract infection developed in each of the 3 animals. Clinical signs of the MERS-CoV infection were benign, but each of the camels shed large quantities of virus from the upper respiratory tract. We detected infectious virus in nasal secretions through 7 days postinoculation, and viral RNA up to 35 days postinoculation. The pattern of shedding and propensity for the upper respiratory tract infection in dromedary camels may help explain the lack of systemic illness among naturally infected camels and the means of efficient camel-to-camel and camel-to-human transmission.

## Abstract 2

Camels infected with MERS-CoV show few symptoms and likely transmit the virus to humans and other camels through respiratory secretions.

The Middle East respiratory syndrome coronavirus (MERS-CoV) was first recognized in 2012 related to a fatal human case of pneumonia in Saudi Arabia (*1*). Currently, >800 cases of MERS have been identified, and the estimated case-fatality rate is ≈35% (*2*). Most cases have been identified on the Arabian Peninsula, but several travel-associated cases have been reported (*2*–*4*). Human-to-human transmission has been reported, predominantly among persons in health care facilities and households; the rate of human infection by zoonotic transmission from a reservoir source is currently not known (*4*–*6*).

The close phylogenetic relationship of human MERS-CoV isolates with those obtained from bats initially suggested a direct link between the emergence of MERS-CoV and a putative natural reservoir (*7*–*9*). Anecdotal reports mentioned contact of MERS-CoV–infected patients with camels and goats, suggesting that livestock might be the intermediate reservoir host for MERS-CoV (*4*,*10*–*12*). Serologic studies revealed widespread prevalence of MERS-CoV–specific antibodies in dromedary camels from several countries that reported MERS cases (*4*,*13*–*19*). Further, MERS-CoV RNA was detected in nasal swab samples obtained from 3 camels on a farm linked to 2 human MERS-CoV cases, and the virus was isolated from nasal swab samples from dromedary camels in Qatar (*14*). MERS-CoV isolation and subsequent full genome sequencing directly linked a dromedary camel and a fatal MERS-CoV case in a person in Saudi Arabia (*20*,*21*). Despite these associations, the role of camels as a primary reservoir for MERS-CoV is still debated (*22*,*23*). Here we report on the experimental inoculation of 3 camels with a human isolate of MERS-CoV.

## Materials and Methods

### Virus and Cells

MERS-CoV (strain HCoV-EMC/2012) was provided by the Department of Viroscience, Erasmus Medical Center, Rotterdam, The Netherlands. The virus was propagated in Vero E6 cells cultured in Dulbecco modified Eagle medium (Invitrogen, Carlsbad, CA, USA) supplemented with 2% fetal bovine serum, 2 mmol/L glutamine, 50 U/mL penicillin, and 50 μg/mL streptomycin.

### Animal Study

Three native-born adult male dromedary camels (*Camelus dromedarius*) were obtained through private sale; the animals tested negative by neutralization assay for MERS-CoV and for bovine coronavirus by ELISA. Camels 1, 2, and 3 were 2, 3, and 5 years old, respectively. Camels 1 and 2 were intact males, and camel 3 had been castrated. Animals were housed in an Animal Biosafety Level 3 facility for the duration of the experiment and fed ad libitum. Camels were acclimated to the facility for 2 weeks before virus inoculation. We sedated the camels with xylazine, then inoculated them with a total dose of 10^7^ 50% tissue culture infective dose (TCID_50_) of MERS-CoV (strain HCoV-EMC/2012) in a total volume of 15 mL, by way of intratracheal (8 mL using transcutaneous catheter), intranasal (3.3 mL in each nostril by expulsion from a syringe), and conjunctival (0.2 mL in each conjunctival sac) routes. The routes of inoculation and infectious dose were chosen to reflect a combination of most likely routes of exposure and to increase the potential of infection. The animals were observed at least 1× daily for the duration of the experiment for behavior, food consumption, activity level, and nasal discharge. Rectal temperature was taken daily from 2 to 7 days postinoculation, then 3× weekly until the animals were euthanized. Nasal and oral swab samples and fecal samples were collected into virus transport medium or virus lysis buffer daily from 0 to 7 days postinoculation (dpi), then 3× weekly until the animal was euthanized. Blood was collected into evacuated EDTA and serum-separating tubes daily at 0–7 dpi and 3× weekly thereafter. Urine was collected by convenience and at necropsy. To evaluate whether virus is exhaled from infected camels, a funnel was placed over the muzzle of each camel and connected to a vacuum pump to capture exhaled air in tissue culture media (10 mL Dulbecco modified Eagle medium, 1% fetal bovine serum, 0.013% SE-15 (anti-foam) with an All Glass Impinger (Ace Glass Inc., Vineland, NJ, USA). Exhaled breath was collected for ≈2 minutes and analyzed by quantitative real-time PCR (qPCR) and virus titration. On days 5, 28, and 42, camels 1, 2, and 3, respectively, were euthanized, and samples were collected from nasal turbinates, lungs, trachea, larynx, pharynx, liver, spleen, kidney, bladder, urine, duodenum, jejunum, colon, rectum, abomasum, forestomachs, prescapular lymph node, retropharyngeal lymph node, tracheobronchial lymph node, mediastinal lymph node, mesenteric lymph node, medulla, and olfactory cortex.

### RNA Extraction and Quantitative PCR

We extracted RNA from swab samples, fecal samples, and serum samples using the QIAamp Viral RNA kit (QIAGEN, Valencia, CA, USA) according to the manufacturer’s instructions. For detection of viral RNA, we used 5 μL of RNA in a one-step real-time reverse transcription PCR upE assay (*24*) using the Rotor-GeneTM probe kit (QIAGEN) according to manufacturer’s instructions. Standard dilutions of a titered virus stock were run in parallel, to calculate TCID_50_ equivalents in the samples (*25*).

### Virus Titration and Plaque Reduction Neutralization Test

We titrated swab samples in viral transport medium, whole blood, and homogenized tissues (≈10% wt/vol) for MERS-CoV virus by plaque assay. Briefly, 10-fold serial dilutions of samples were prepared in BA-1 medium (MEM, 1% bovine serum albumin, 350 mg/L sodium bicarbonate, 50 mM Tris, pH 7.6, 5 mg/L phenol red) containing 100 mg gentamicin, 200,000 U penicillin G, 100 mg streptomycin, and 5 mg amphotericin/L; plaque assay was conducted as described for West Nile virus (*26*). Plaques were counted on days 1 and 3 after the second overlay and virus titers were expressed as PFUs per mL or gram. We determined neutralizing antibody titers by plaque reduction neutralization test as described (*26*), using a 90% neutralization cutoff.

### Histopathologic Examination and Immunohistochemical Testing

Tissues were fixed for >7 days in 10% neutral-buffered formalin and embedded in paraffin. Tissue sections were stained with hematoxylin and eosin. To detect MERS-CoV antigen, we completed immunohistochemical testing using a rabbit polyclonal antiserum against HCoV-EMC/2012 (1:1,000) as a primary antibody.

## Results

### Clinical Signs in Dromedary Camels Inoculated with MERS-CoV

**Figure 1:**
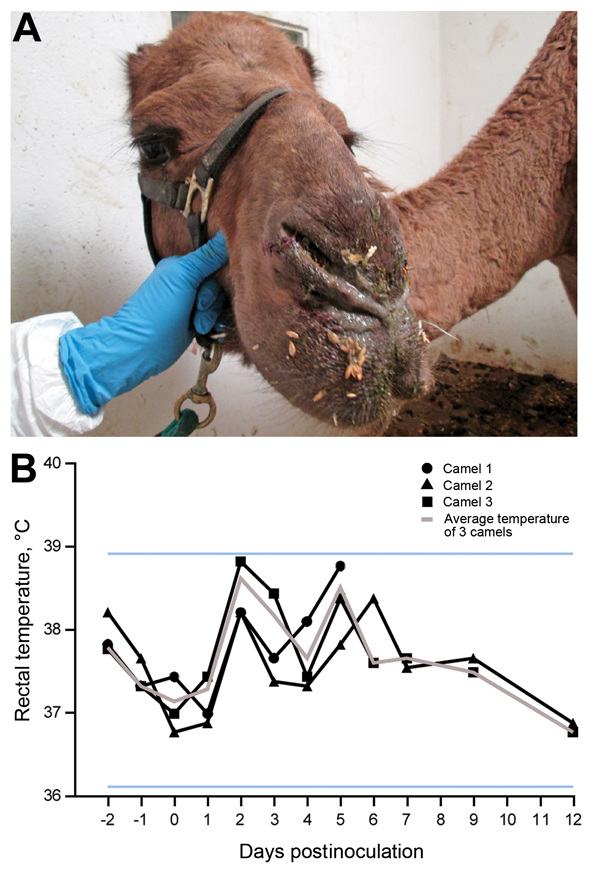
Clinical signs in dromedary camels inoculated with Middle East respiratory syndrome coronavirus (MERS-CoV). A) Nasal discharge observed in camel 3; each of 3 inoculated camels had nasal discharge during the first 2 weeks of the experiment. B) Rectal temperatures are indicated for each camel by lines with geometric shapes. Horizontal lines indicate the normal temperature range observed among these dromedary camels as calculated by mean ± 3×, the before inoculation.

Each camel showed minor clinical signs of disease, consisting of rhinorrhea (Figure 1, panel A) and a mild elevation in body temperature at 2 dpi and 5–6 dpi (Figure 1, panel B); no other clinical signs were observed. Rhinorrhea developed in all 3 camels beginning at 2 (camels 1 and 3) and 5 (camel 2) dpi, and persisted <2 weeks. The nasal discharge drained from both nares and varied in character from serous to purulent; minor hemorrhage was observed on some occasions, but may have been caused by trauma that occurred during collection of samples.

### MERS-CoV Shedding

**Figure 2:**
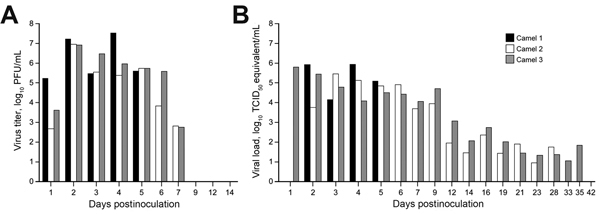
Virus shedding from the upper respiratory tract in dromedary camels inoculated Middle East respiratory syndrome coronavirus (MERS-CoV). Shedding was determined by A) infectious titers by plaque assay and B) viral load by quantitative real-time PCR. We extrapolated 50% tissue culture infective dose (TCID_50_) equivalents from standard curves generated by 10-fold dilutions of a MERS-CoV stock (HCoV-EMC/2012) with known virus titer in parallel to each quantitative real-time PCR run.

**Figure 3:**
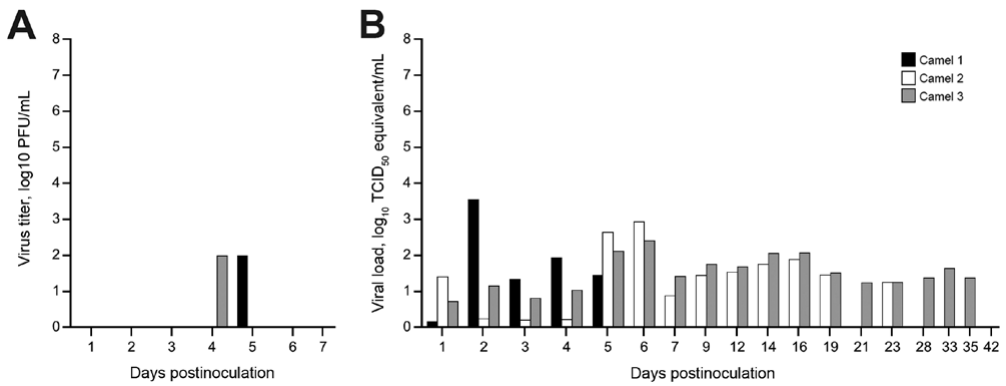
Virus shedding determined by oral swabs obtained from dromedary camels inoculated with Middle East respiratory syndrome coronavirus (MERS-CoV). Shedding was determined by A) determining infectious titers and B) viral RNA. Virus titers were determined by plaque assay and viral load by qRT-PCR. TCID_50_ equivalents were extrapolated from standard curves generated by 10-fold dilutions of a MERS-CoV stock (HCoV-EMC/2012) with known virus titer in parallel to each run.

MERS-CoV shedding started during 1–2 dpi, as detected by the presence of infectious virus and viral RNA by qPCR in nasal swab samples. Infectious virus shedding was detected <7 dpi, and shedding of viral RNA was detected <35 dpi in nasal swab samples (Figure 2). Low concentrations of infectious virus and viral RNA were detected in oral samples, likely originating in drainage from the nasal cavity (Figure 3). No viral RNA was detected in fecal samples or in urine samples collected by convenience or at necropsy at 0, 1, 5, 14, 21, 28, and 42 dpi from the 3 camels. No infectious virus or viral RNA was detected in any of the serum or whole blood samples. Small quantities of MERS-CoV RNA were detected in exhaled breath by qPCR (10^1.2^ and 10^1.4^ TCID_50_ equivalent/mL) at 3 and 5 dpi, but infectious virus was not detected.

### MERS-CoV Tropism and Pathology

**Figure 4:**
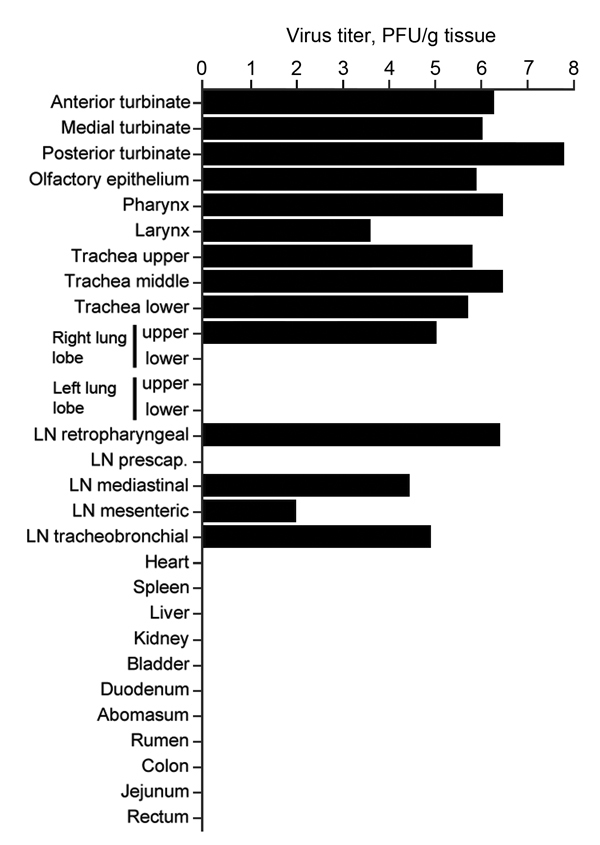
Virus titers in tissues collected from dromedary camel 1 inoculated with Middle East respiratory syndrome coronavirus (MERS-CoV). Tissues were collected at 5 days postinoculation (dpi) for camel 1, 28 dpi for vamel 2 and 42 dpi for camel 3. Detectable infectious virus in the collected tissues was found only in camel 1. Nasal turbinates were sampled in 3 different sections: anterior, medial, and posterior. Infectious titers were determined by plaque assay. LN, lymph node.

**Figure 5:**
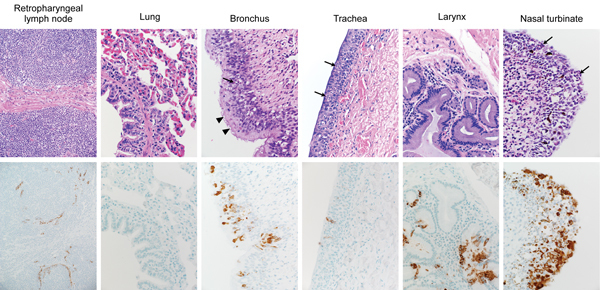
Histopathologic changes at 5 days postinoculation in camel 1 inoculated with Middle East respiratory syndrome coronavirus (MERS-CoV). Tissues were collected and stained with hematoxylin and eosin (top row). Anti–MERS-CoV immunohistochemical results (bottom row) are visible as a red-brown stain. Degeneration of the pseudostratified epithelium lining the nasal turbinate, trachea, and bronchus is indicated by the absence of goblet cells, cilia and nuclear regimentation with infiltration of neutrophils (arrows). The arrowheads indicate areas where the cilia remained intact. Original magnification ×400.

Infectious virus was detected in tissues from camel 1, which was euthanized on 5 dpi, but not in tissues obtained from camels 2 and 3, which were euthanized at28 and 42 dpi, respectively. Infectious virus was detected in tissues of the upper respiratory tract (URT), including nasal turbinates, olfactory epithelium, pharynx, and larynx. In the lower respiratory tract, infectious virus was detected in the trachea and in 1 of 4 lung lobes tested. Infectious virus was also detected in the retropharyngeal, mediastinal, mesenteric, and tracheobronchial lymph nodes (Figure 4). On necropsy of camel 1 at 5 dpi, histologic lesions were found in the pseudostratified epithelial cells in the URT and the lower respiratory tract (trachea, bronchi, and bronchioles) but not in the alveoli (Figure 5). The lesions were characterized as mild to moderate acute intraepithelial and submucosal inflammation with multifocal necrosis and loss of pseudostratified epithelial cells, comparable to the common cold among humans. Multifocal loss of epithelial polarity and cilia with squamous metaplasia were observed. The epithelium was infiltrated by small-to-moderate numbers of neutrophils with fewer macrophages; similar inflammatory cells also permeated the submucosa. The submucosal glands of the trachea were multifocally necrotic and infiltrated by small numbers of neutrophils. Viral antigen was detected within the epithelial cells of the nasal turbinates, larynx, trachea, bronchi, and bronchioles, but not the alveoli. In addition, viral antigen was present at the follicular mantle zone of the tonsils and mediastinal and retropharyncheal lymph nodes (Figure 5). The nasal turbinates, larynx, and trachea of camel 2 (necropsied at 28 dpi) had similar but milder lesions when compared with those of camel 1. The nasal turbinate, larynx, and bronchus showed small numbers of infiltrating neutrophils; however, in contrast with the condition of camel 1, the cilia and goblet cells were intact. The remainder of the respiratory tract of camel 2 was unaffected. Immunohistochemical testing revealed the presence of limited viral antigen in the nasal turbinate but not in any of the other tissues at that time. No lesions or viral antigens were detected in camel 3 at 42 dpi.

### Humoral Response to MERS-CoV

Serum samples were collected weekly from the camels to monitor the generation of neutralizing antibodies specific to MERS CoV. Each of the 3 camels was seronegative before inoculation. Robust MERS-CoV specific antibody responses developed in camels 2 and 3 (euthanized on 28 and 42 dpi, respectively), detected first on 14 dpi with a plaque-reduction neutralization test titer from 20 to 40 that increased to 640 at 35 dpi (Table). Camel 1 was euthanized at 5 dpi and was not tested for development of antibodies against the virus.

**Table:** Antibody titers against MERS-CoV in dromedary camels inoculated with the virus as determined by 90% plaque reduction assay*

Day	Camel 1	Camel 2	Camel 3
0	<10	<10	<10
7	NA†	<10	<10
14	NA	40	20
21	NA	80	20
28	NA	40	160
35	NA	NA	640
42	NA	NA	320

*MERS-CoV, Middle East respiratory syndrome coronavirus. †NA, serum samples were not available.

## Discussion

Epidemiologic and surveillance data on the emergence of MERS-CoV strongly point toward a role for dromedary camels as a reservoir for zoonotic transmission (*13*–*21*,*27*,*28*). To understand the ecology of MERS-CoV in the most likely reservoir host, we experimentally inoculated 3 young adult dromedary camels with MERS CoV. The disease observed was clinically benign, in agreement with the absence of overt illness reported from field surveillance studies (*14*,*17*,*19*,*21*). A large quantity of MERS-CoV and viral RNA was detected in nasal swab specimens from each of the 3 camels. Infectious virus was detected through 7 dpi, and RNA was detected through 35 dpi in camel 3, which was euthanized on day 42. This route of shedding is consistent with data on naturally infected camels (*14*,*18*,*21*,*28*,*29*), and the pattern of shedding suggests that the infectious period of camels may be short. MERS-CoV was not detected in either urine or feces, again consistent with field observations (*21*,*28*).

The large quantities of MERS-CoV shed in nasal secretions by each of the 3 camels suggest that camel-to-camel and camel-to-human transmission may occur readily through direct contact and large droplet, or possibly fomite transmission. Histopathologic examination revealed that the URT, specifically the respiratory epithelium in the nasal turbinates, is the predominant site of MERS-CoV replication in camels.

Neutralizing antibodies were detected from 14 dpi onward, reaching a maximum neutralizing titer of 640 after 35 days. Serologic studies in camels in the field have reported MERS-CoV neutralizing titers as high as 5,120 (*14*,*16*), potentially indicative of repeated exposure and re-infection.

The study reported here was done on the basis of inoculation of 3 male animals with a human isolate of MERS-CoV, and the study design we used imposed several limitations on how these data inform what occurs in natural infections. The camels we inoculated were exposed to a high dose of virus by 3 simultaneous routes of inoculation. In retrospect, the inoculation dose does not seem excessive, based on the large quantity of virus shed nasally in all 3 animals (Figure 2). The total dose inoculated was relatively equivalent to the amount of virus present in a single nasal swab sample taken during the first days postinoculation, and it seems probable that a camel shedding this quantity of virus would readily infect other camels or humans with which it had direct contact. The fact that we inoculated the camels with the virus by 3 routes precludes drawing conclusions regarding efficiency of transmission by a particular route, which is a topic that should be addressed in future studies. The influence of camel age on susceptibility and dynamics of virus shedding is another notable parameter that requires further study. It seems likely that productive infection and shedding of virus in natural settings occurs predominantly in juvenile camels (*28*). This could be the result of an intrinsic difference in age-related susceptibility, but is more likely related to the immunologically naïve status of the animals in the context of a high force of infection after decay of passively acquired antibodies. The animals we infected were young adults, but were seronegative and therefore probably as susceptible as juveniles from MERS-CoV–endemic regions. Another aspect of pathogenesis not addressed here is whether virus is present in milk or meat from infected camels and thereby poses another potential route of exposure to humans who consume such products. Despite these limitations, the magnitude and pattern of virus shedding was essentially identical in all 3 animals and supports the available epidemiologic data indicating that camels are likely a major reservoir host for MERS-CoV. Additional experimental and field studies are clearly required to address the duration of shedding of infectious MERS-CoV from infected camels, to determine whether infection results in protective immunity, and to clarify the burden of illness among humans resulting from transmission from camels.
